# Residual positioning errors and uncertainties for pediatric craniospinal irradiation and the impact of image guidance

**DOI:** 10.1186/s13014-020-01588-2

**Published:** 2020-06-10

**Authors:** Daniel Gram, André Haraldsson, N. Patrik Brodin, Karsten Nysom, Thomas Björk-Eriksson, Per Munck af Rosenschöld

**Affiliations:** 1 Department of Oncology – Section of Radiotherapy, Rigshospitalet, Belgdamsvej 9, DK-2100 Copenhagen, Denmark; 2 Niels Bohr Institute, University of Copenhagen, Copenhagen, Denmark; 3 Radiation Physics - Department of Hematology, Oncology and Radiation Physics, Skåne University Hospital, Lund, Sweden; 4 Department of Clinical Sciences, Lund University, Medical Radiation Physics, Lund, Sweden; 5 Institute for Onco-Physics, Albert Einstein College of Medicine and Montefiore Medical Center, Bronx, NY USA; 6 Department of Paediatrics and Adolescent Medicine, The Juliane Marie Center, Rigshospitalet, Copenhagen, Denmark; 7 Department of Oncology, Institute of Clinical Sciences, Sahlgrenska Academy at the University of Gothenburg, Gothenburg, Sweden; 8 Regional Cancer Centre West, Gothenburg, Sweden

**Keywords:** Positioning errors, Positioning uncertainties, Residual setup errors, Craniospinal irradiation, Image guidance

## Abstract

**Background:**

Optimal alignment is of utmost importance when treating pediatric patients with craniospinal irradiation (CSI), especially with regards to field junctions and multiple isocenters and techniques applying high dose gradients. Here, we investigated the setup errors and uncertainties for pediatric CSI using different setup verification protocols.

**Methods:**

A total of 38 pediatric patients treated with CSI were identified for whom treatment records and setup images were available. The setup images were registered retrospectively to the reference image using an automated tool and matching on bony anatomy, subsequently, the impact of different correction protocols was simulated.

**Results:**

For an action-level (AL)-protocol and a non-action level (NAL)-protocol, the translational residual setup error can be as large as 24 mm for an individual patient during a single fraction, and the rotational error as large as 6.1°. With daily IGRT, the maximum setup errors were reduced to 1 mm translational and 5.4° rotational versus 1 mm translational and 2.4° rotational for 3- and 6- degrees of freedom (DoF) couch shifts, respectively. With a daily 6-DoF IGRT protocol for a wide field junction irradiation technique, the residual positioning uncertainty was below 1 mm and 1° for translational and rotational directions, respectively. The largest rotational uncertainty was found for the patients’ roll even though this was the least common type of rotational error, while the largest translational uncertainty was found in the patients’ anterior-posterior-axis.

**Conclusions:**

These results allow for informed margin calculation and robust optimization of treatments. Daily IGRT is the superior choice for setup of pediatric patients treated with CSI, although centers that do not have this option could use the results presented here to improve their margins and uncertainty estimates for a more accurate treatment alignment.

## Background

Second only to leukemia, primary tumors in the central nervous system (CNS) are the most common malignancies in children [1]. The treatment usually consists of surgery, chemotherapy and irradiation, depending on age and tumor-related risk factors. When treating pediatric patients with CNS tumors it is of utmost importance that the patients are optimally aligned since this anatomical region contains many organs-at-risk (OARs) and since the developing brain is particularly vulnerable to the long-term toxicities of radiotherapy. Recently, studies investigating hippocampal-sparing cranial irradiation including craniospinal irradiation (CSI) for patients with medulloblastoma have emerged in order to minimize the common, treatment related, neurocognitive side effects [2, 3]. When trying to avoid an important OAR such as the hippocampus, the importance of accurate alignment become even more apparent.

Setup corrections have typically been based on off-line setup images obtained from skin-mark based positioning protocols including different action level (AL)-protocols and non-action level (NAL)-protocols during the initial fractions of the treatment schedule [4, 5]. Recently, setup correction decisions have changed from being based on AL/NAL-protocols to daily pre-treatment image-guided radiotherapy (IGRT) [6].

Setup uncertainties have been extensively studied in photon radiotherapy for various treatment sites [7–20]. Lately, proton radiotherapy has emerged as a prominent alternative to photon therapy for pediatric CSI and today, both treatment modalities are relevant when studying residual errors and uncertainties. For example, as setup errors will result in different dose distributions for photon treatments, they may cause even worse distortions of the dose distributions for proton therapy, due to the misalignment of the beams and the sensitivity to varying tissue densities [21, 22].

In this multicenter study we investigated the setup errors for pediatric patients undergoing CSI by following image-guided correction protocols and explored how AL/NAL-protocols and daily IGRT impact the positioning uncertainty. These positioning uncertainty data may be used to estimate an uncertainty budget available for planning target volumes (PTV) and OAR margins as well as estimating criteria for robust optimization [23], which are essential components for the safe implementation of CSI for pediatric patients.

## Methods

All patients ≤20 years at the time of treatment who received CSI (Childhood centers, oncology and radiotherapy departments from both Denmark and Sweden) between 2005 and 2018 were reviewed in accordance with approval from The Danish Patient Safety Authority and The Danish Data Protection Agency. A total of 38 eligible patients were identified, for whom treatment records and setup images (a minimum of the first four consecutive fractions were required for inclusion, up to all 20 fractions) were available, and included in the analysis (Table 1 provides the patients’ characteristics).

**Table 1:** Characteristics of the 38 pediatric and adolescent patients included in the study. The number of fractions was 13 or 20 depending on tumor-related risk factors and are prescribed 1.8 Gy / fraction to either 23.4 Gy or 36 Gy to the craniospinal volume

	Median	Range
Age (y)	8	4–19
Sex	**n**	**%**
Male	25	65.8
Female	13	34.2
Position		
Supine	35	92.1
Prone	3	7.9
Treatment fractions		
13	30	78.9
20	8	21.1
Anesthesia		
Yes	25	65.8
No	13	34.2
Isocenters		
1	20	52.6
2	6	15.8
3	12	31.6
Treatment unit		
Linac	18	47.4
3D-CRT	15	83.3
kV	13	86.7
MV	2	13.3
IMRT	3	16.7
kV	3	100
Tomo	20	52.6
Disease		
Medulloblastoma	21	55.3
Ependymoma	3	7.9
Germinoma	2	5.3
Astrocytoma gr. 2	2	5.3
Other	9	23.6
Unknown	1	2.6
	**Mean**	**SD**
Field length (cm)	65.2	10.7
BMI (Z-score)	0.07	2.0

Abbreviations: *BMI* Body mass index, *Tomo* Tomotherapy, *Linac* Linear accelerator

The pediatric patients were setup according to the clinical procedure using image verification, in which only the three-dimensional couch corrections (“3- degrees of freedom, DoF”) were used for positioning these patients during treatment, ignoring the rotational deviations. The most common patient immobilization was a full body vacuum bag with a head mask together with a mouthpiece. Over the years, the immobilization was slightly adjusted since the patients were treated over the course of 13 years. The patients were aligned to wall mounted lasers followed by x-ray images taken at each isocenter and a shift was applied using a mean correction based on the images. New images were taken before the treatment at each isocenter. For the tomotherapy unit, a single full body scan was used for positioning of the patients.

For the current study, the setup images used for positioning were reanalyzed in order to estimate the set-up uncertainty of the patients, according to previously published methods; van Herk [24] and Kutcher et al. [7]. The positioning deviations quantified using the image data may be small and a correction may have been deemed unnecessary to perform clinically. We reexamined all the setup images and they were retrospectively registered to the reference image(s) using an automatic matching procedure based on bony anatomy. The match box volume of interest was set to cover the cranium, and the first two cervical vertebrae, ignoring as much as possible of the chin for the cranial isocenter while for the thoracic and lumbar isocenters, the spine was covered, omitting the top and bottom vertebrae. For the tomotherapy unit, the volume of interest was focused around the isocenter (thoracic region), however still trying to match the entire craniospinal volume. The different image modalities used were mega-voltage computed tomography (MVCT) for Tomotherapy and either kilo-voltage (kV) cone-beam computed tomography (CBCT) or planar kV/MV images using the on-board imaging device or electronic portal imaging device, respectively, for linear accelerators (Offline Review – multi-modality image review, ARIA™ Oncology Information System v. 13.7, Varian medical systems, Palo Alto, CA, USA and CTrue™, Accuray Inc., Madison, WI, USA). A 3- and 6-DoF match was performed, respectively, using both translational (superior-inferior (SI), anterior-posterior (AP) and medial-lateral (ML)) and rotational (yaw = rotation around the AP axis, pitch = rotation around the ML axis and roll = rotation around the SI axis) information. Using the image registration procedure, we calculated the mean correction, residual error and standard deviation (SD) for each patient. The mean correction is defined as the correction used in AL -protocols while the residual error is the mean discrepancy between the clinically applied and ideal registrations (found through retrospective matching) for all fractions for a single patient. Similar to van Herk [24] and Kutcher et al. [7], we used the data available to derive the systematic error (SE), systematic uncertainty (SU) and random uncertainty (RU) for all patients. The SE was calculated by taking the average mean residual error for all patients over their entire treatment and should thus be close to zero unless there is a systematic deviation affecting the procedure (e.g. misaligned lasers or similar). The SU and RU were calculated through the SD of the mean errors for all patients and the root mean square of the SD for all patients, again over the entire treatment, respectively.

Patient characteristics analyzed included the total length of the treatment field, body mass index (BMI, calculated at the start of treatment), age at treatment, sex, patient positioning (prone or supine), number of isocenters (these are associated with treatment modality, Tomotherapy patients had one isocenter while patients treated on linear accelerators had multiple isocenters) and whether the patient was treated under general anesthesia or not (Table 1). The majority of the younger aged (<10y) children were treated with a single isocenter. Since BMI of children and adolescents varies considerably with sex and age, the BMI was expressed as Z-scores [25], calculated according to previously published methods [26, 27].

Using the positioning uncertainty data, we simulated four image guidance correction protocols; (1) an AL (based on the first three fractions with online corrections, followed by an isocenter shift according to the average deviation), (2) a NAL (based on the first three fractions without online corrections, followed by an isocenter shift according to the average deviation), (3) daily IGRT protocol for narrow field junctions (nj) and (4) daily IGRT for wide field junctions (wj). Each protocol was simulated for image guidance with a 3-DoF and 6-DoF couch. We refer to “nj” as a treatment protocol with narrow field junctions and sharp dose gradients, i.e. where the field positions cannot be altered in the cranio-caudal direction without the risk of introducing considerable hot- or cold-spots in the dose distribution. For this protocol, no change in longitudinal position was allowed between isocenters. The “wj” protocol refers to the situation where wide field junctions and flat dose gradients are optimized to be overlapping, thus, dosimetric consequences of uncertainties in the cranio-caudal directions will be very small. For example, a narrow field junction can have a sharp dose gradient corresponding to 5% of the prescribed dose / mm deviation in the SI direction which corresponds to 1.8 Gy for a prescribed dose of 36 Gy with only a single millimeter misalignment. The flat dose gradient emanating from the wide field junction may have the equivalent of around 0.6% / mm deviation. The wide field junctions and flat dose gradients are usually obtainable using more modern techniques such as volumetric modulated arc therapy (VMAT) and intensity modulated proton therapy (IMPT), while the narrow junctions and sharp dose gradients are the result of three-dimensional conformal radiotherapy. Consequently, all available degrees of freedom were applied for this protocol. All simulations were performed based on the protocols previously described where all relevant shifts and corrections were applied to the images before the residual errors were assessed and the uncertainties were subsequently calculated.

### Statistical analysis

The normality and linearity assumptions for the association between patient characteristics and residual errors were tested with Shapiro-Wilk tests and visual inspection of histograms and scatter plots. Data for positioning uncertainties for the different image-guided protocols were evaluated against pre-treatment image setup data and univariate linear regression models were fitted for the various positioning uncertainties and residual errors using all covariates. Bivariate associations between all patient characteristics (age, sex, position, anesthesia, number of isocenters, field length and BMI) and the positioning uncertainties and residual errors where quantified with Spearman’s rank correlation coefficients or Wilcoxon’s rank-sum tests for continuous and categorical variables, respectively.

Since the variance of each isocenter for all cardinal directions is assumed to be the same (based on a two-sample F-test that did not reject the null hypothesis that the samples comes from normal distributions and the same variance (*p* = 0.054–0.799)), this data is pooled to increase the statistical power of the comparison.

## Results

### Residual setup errors

The residual errors should only include rotational deviation since translational errors were corrected at treatment. However, rotational errors can affect the translational deviation as well. The SE was found to be well below 0.1 mm in all cardinal directions, for both 3-DoF and 6-DoF for the pooled data.

Translational positioning deviations greater than 1 cm occurred in 6% of all fractions and 33% of the patients had at least one such correction while rotational deviations greater than 1° occurred in 34% of all fractions and 80% of the patients had at least one such correction. The majority of the residual setup errors were found for the lumbar isocenter. Every patient in this study had at least one deviation larger than the PTV margin (SI = 10 mm, AP = 12 mm, ML = 18 mm) used for these patients and constituted therefore a geometric miss for all patients treated, not using a daily IGRT-protocol.

With an AL/NAL-protocol, the translational residual setup error was found to be as high as 2.4 cm for an individual patient during a single fraction, and the rotational error as high as 6.1°. If using daily IGRT the maximum setup error was reduced to 0.1 cm translational and 5.4° rotational and 0.1 cm translational and 2.4° rotational setup error for 3- and 6-DoF couch shifts, respectively (using maximum allowed pitch and roll correction of 3°).

There were no statistically significant correlations between the residual setup errors with gender and setup (prone/supine) position. We found moderate to strong positive correlations for total field length (*r* = 0.5 *p* = 0.04) and (*r* = 0.6 *p* < 0.001) for residual setup error and standard deviation, respectively, i.e. a longer total field length correlated with a larger residual setup error and standard deviation. For Linac-based multiple isocenter treatments, this presents an issue for standardizing margins where corrections in the SI direction cannot be applied after the first isocenter(s) position has been treated. The IGRT (nj) protocol eliminated correlations in all directions except SI while the IGRT (wj) protocol eliminated all significant correlations and relationships. Fewer isocenters were correlated with a lower mean residual setup error.

### Setup uncertainties

**Fig. 1:**
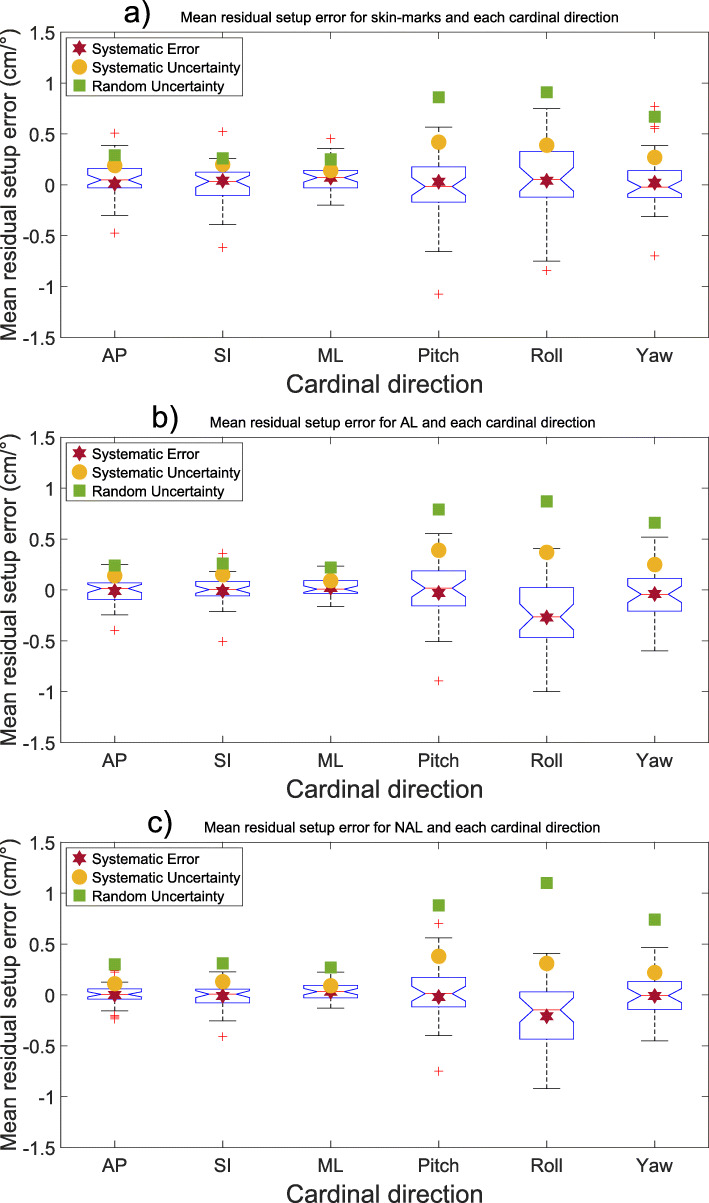
Mean setup error (mm) presented with blue notched boxplots for (**a**) skin-marks, (**b**) AL-protocol and (**c**) NAL-protocol and all six cardinal directions examined. The boxplots show the median (central red line), 25th and 75th percentile (blue notched box) and the whiskers (black dashed lines) which extend to the most extreme data points that are considered non-outliers. The individually plotted red plus signs indicate the outliers. Please note that the plots are showing two different dimensions (cm and °)

**Fig. 2:**
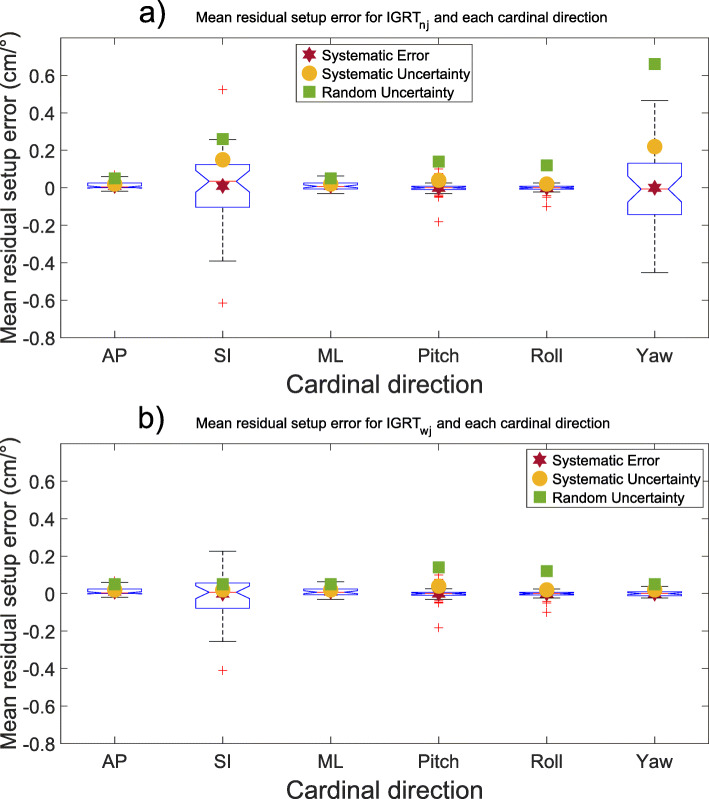
Mean setup error (mm) presented with blue notched boxplots for (**a**) IGRT (nj)-protocol and (**b**) IGRT (wj)-protocol and all six cardinal directions examined. The boxplots show the median (central red line), 25th and 75th percentile (blue notched box) and the whiskers (black dashed lines) which extend to the most extreme data points that are considered non-outliers. The individually plotted red plus signs indicate the outliers. Please note that the plots are showing two different dimensions (cm and °)

When correcting the shifts according to any of the imaging protocols, large inter-fractional deviations occurred especially for rotational deviations (the uncertainties presented in Table 2 and Figs. 1 and 2 illustrates the tendencies, with a larger uncertainty for larger deviations). The uncertainties for the pooled isocenters and all cardinal directions for all imaging protocols are presented in Table 2 and Figs. 1 and 2. The largest rotational uncertainty was found for the patients’ roll, even though this was the least common type of rotational error, while the largest translational uncertainty was found in the patients’ AP-axis.

**Table 2:** Systematic uncertainty (SU), as calculated by the mean, and random uncertainty (RU), as calculated by the root mean square deviation, for all imaging protocols, both 3- and 6- degrees of freedom (DoF) and all isocenters pooled (Units: cm and °/degrees). Bold numbers indicate statistically significant difference compared to skin-mark based setup

3DoF	SI		AP		ML	
	SU	RU	SU	RU	SU	RU
Translational						
- IGRT (nj)	0.18	0.26	**0.03**	**0.05**	**0.03**	**0.05**
- IGRT (wj)	**0.02**	**0.05**	**0.03**	**0.05**	**0.03**	**0.05**
- Skin	0.20	0.27	0.18	0.27	0.12	0.23
- AL	0.20	0.26	0.13	0.22	0.07	0.20
- NAL	0.18	0.32	0.09	0.28	0.07	0.24
6DoF	SI/Roll		AP/Yaw		ML/Pitch	
	SU	RU	SU	RU	SU	RU
Translational						
- IGRT (nj)	0.15	0.26	**0.02**	**0.05**	**0.02**	**0.05**
- IGRT (wj)	**0.02**	**0.05**	**0.02**	**0.05**	**0.02**	**0.05**
- Skin	0.20	0.26	0.19	0.31	0.14	0.27
- AL	0.15	0.26	0.14	0.26	0.09	0.24
- NAL	0.13	0.31	0.11	0.32	0.09	0.29
Rotational						
- IGRT (nj)	**0.02**	**0.12**	0.22	0.66	**0.04**	**0.14**
- IGRT (wj)	**0.02**	**0.12**	**0.02**	**0.05**	**0.04**	**0.14**
- Skin	0.39	0.91	0.27	0.67	0.42	0.86
- AL	0.37	0.87	0.25	0.66	0.39	0.79
- NAL	0.31	1.10	0.22	0.74	0.38	0.88

Abbreviations: *DoF* Degrees of freedom, *SI* Superior-inferior, *AP* Anteroposterior, *ML* Medial-lateral, *SU* Systematic uncertainty, *RU* Random uncertainty, *IGRT* Image-guided radiotherapy, *nj* Narrow field junction, *wj* Wide field junction, *AL* Action level, *NAL* Non-action level

There were no statistically significant correlations between uncertainties with gender and setup position. We found that a higher BMI correlated with a larger SU in the SI direction (*r* = 0.35–0.46, *p* = 0.008–0.04) but not in the other cardinal directions. The number of isocenters, age and anesthesia showed weak to moderate correlations (*r* = − 0.63 – 0.45, *p* = 0.008–0.02). Younger children are usually treated under general anesthesia and we found that being under general anesthesia could reduce the setup uncertainties in the SI direction since there were smaller deviations for these patients (*r* = − 0.39 – − 0.19, *p* = 0.02–0.46).

If a daily 6-DoF IGRT (wj) protocol was used, the residual systematic positioning uncertainty was 0.2–0.3 mm and 0.02–0.04° for translational and rotational directions, respectively. The residual random positioning uncertainty was 0.5 mm and 0.05–0.14° for translational and rotational directions, respectively. This is significantly smaller than for the corresponding 1.2–2.0 mm and 0.3–0.4° (*p* = 0.03, based on mean values) systematic uncertainties and 2.3–3.1 mm and 0.7–0.9° (*p* = 0.03) random uncertainties, when using only the skin-marks for setup. Both AL- and NAL-protocols with 6-DoF had lower uncertainties compared to only using skin-marks, but the results were not statistically significant (*p* = 0.06, *p* = 0.41, respectively) with similar results for 3-DoF.

Since the data stem from patients treated over the course of 13 years, both immobilization and imaging strategies have changed throughout. A vacuum bag with a mask and/or a mouthpiece was the most common immobilization type and the immobilization changes were conjecturally inconsiderable. However, the setup images revealed that patients treated in the earlier years were more accurately positioned to the skin-marks compared to the patients treated later in the cohort. No other time-trends were observed.

**Fig. 3:**
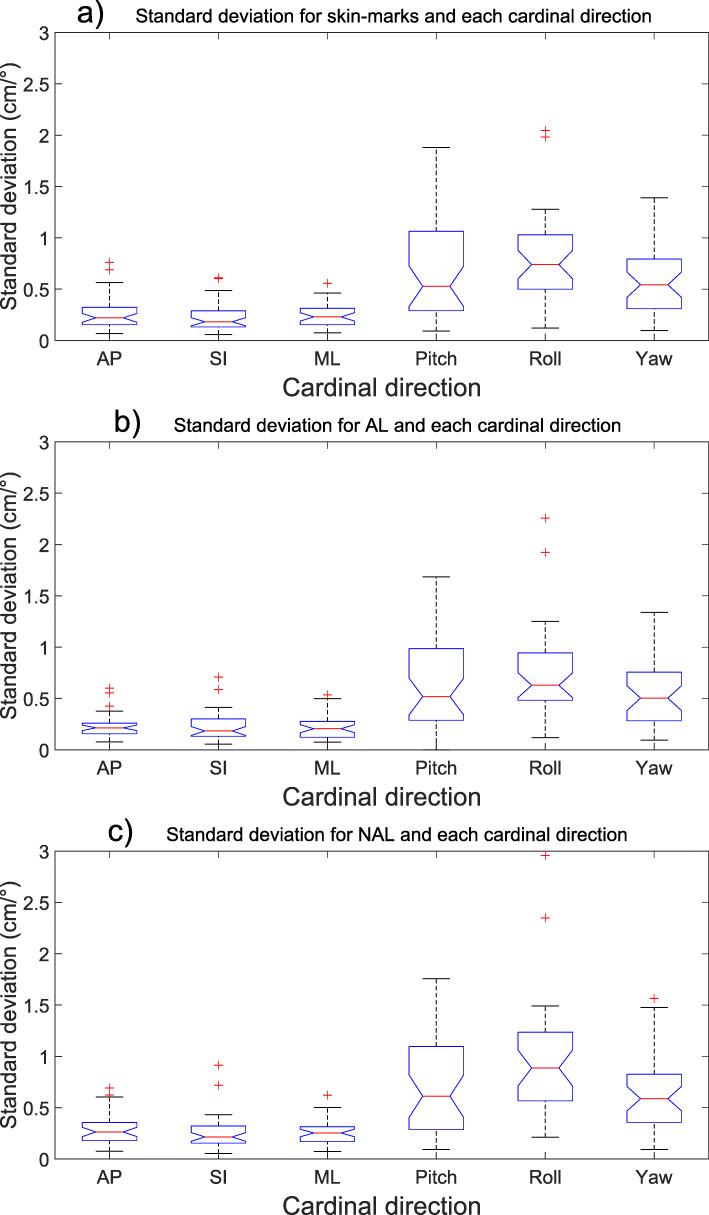
Standard deviation (mm) presented with blue notched boxplots for (**a**) skin-marks, (**b**) AL-protocol and **c)** NAL-protocol and all six cardinal directions examined. The boxplots show the median (central red line), 25th and 75th percentile (blue notched box) and the whiskers (black dashed lines) which extend to the most extreme data points that are considered non-outliers. The individually plotted red plus signs indicate the outliers. Please note that the plots are showing two different dimensions (cm and °)

**Fig. 4:**
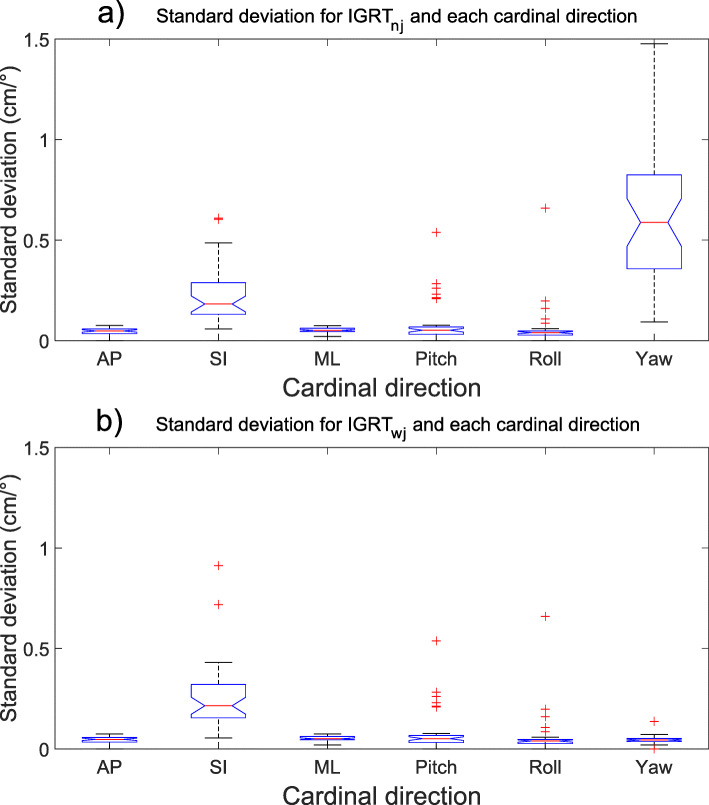
Standard deviation (mm) presented with blue notched boxplots for (**a**) IGRT (nj)-protocol and (**b**) IGRT (wj)-protocol and all six cardinal directions examined. The boxplots show the median (central red line), 25th and 75th percentile (blue notched box) and the whiskers (black dashed lines) which extend to the most extreme data points that are considered non-outliers. The individually plotted red plus signs indicate the outliers. Please note that the plots are showing two different dimensions (cm and °)

Rotational uncertainties are generally more considerable than translational (Table 2 and Fig. 1, 2, 3 and 4), and the effect of rotational uncertainty peaks farthest away from the isocenter and rapidly decreases closer to it. Typically, the largest uncertainties were found to be in the SI direction or around the SI direction (roll). The single largest deviation was found to be 9.6° for the roll rotation around the SI-axis for a NAL-protocol.

Documents for machine quality assurance for the last 5 years were assessed and all radiation isocenter vs imaging isocenter agreements were within 1 mm.

## Discussion

In this study we mainly provide the uncertainties that stem from setup images as there are a wide variety of treatment and imaging units, many with different inherent uncertainties. With these results we hope that clinics providing pediatric CSI will have a possibility to personalize the treatment margins regardless of imaging protocol or number of isocenters in use. Important to note when calculating margins for CSI treatments is that different margin strategies in respect to inter-fractional effects of the organ or structure the margin is based on, e.g. spinal column length, need to be considered. These results could also act as a reference to older methods or when comparing setup verification technique. To the authors’ knowledge, this is the first analysis dealing with positioning uncertainties based solely on pediatric CSI treatments.

According to our results, the random uncertainty increases by using a NAL-protocol to correct for the couch shifts. This could, however, be because the correction merely shifts the scatter of the corrected points (where each point is a patient’s fraction) whilst still using the two starting points (that were not corrected for in a NAL-protocol) when calculating the RU. If the starting points were removed from the calculation, there was a minimal increase in RU by using a NAL-protocol for some directions and isocenters (both pooled and un-pooled data), it was, however, not statistically significant. There is also a small general decrease in RU when removing the starting points which could be an indication that some of the most extreme correction values tend to occur in the first couple of fractions.

It is important to keep in mind that there are two different types of uncertainties with different sources. The random component of the uncertainties is inter-fractional and stem from positioning on external markers on either the patient’s skin or mask or due to internal motion relative to the external markers. The systematic component stems from events such as changes in patient anatomy over the course of treatment or mechanical mismatches between CT simulation and the treatment machine. There can still be quite large errors even if using a daily IGRT-protocol since there is a 3° physical restraint (maximum allowed couch movement in clinical treatment mode) on the couch. Shifts larger than this should trigger a re-positioning of the patient but since we do not have access to the specific circumstances for each treatment fraction, we were restricted to analyzing only the setup images in this study.

Previous studies have developed widely used algorithms for calculating margins [28, 29] and there are multiple alternatives, reported by van Herk [24]. With these algorithms standardized or personalized margins can be calculated. We also wanted to supply information for both narrow- and wide field junctions irradiation techniques, since some centers that use conformal techniques do not allow for imaging-based corrections in the SI direction, and simply apply the planned SI isocenter shift, after treating the first isocenter due to narrow field junctions and steep dose gradients. Most IMPT centers have that option since the wide junctions and more flat dose gradients often result in a smaller dose difference compared to incorrect heterogeneity correction arising from positional errors [21, 23]. This might also explain some of the effects seen in the SI direction. It is important to note that there could be variations in the relative distance between isocenters (Linac patients with multiple isocenters) which can lead to large differences between the expected and actual dose distribution if an IGRT protocol is used to correct the shift in all directions without considerations to the junction. One would also expect that the yaw would contribute largely to the uncertainties in the SI direction, but this is not supported by our results. Hadley et al. [30] studied the effect of a wide single gradient dose junction using intensity modulated radiotherapy for spinal fields which is similar to the technique utilized by many proton centers. They found that this improved uncertainties for spinal fields compared to narrow multiple junction shifts. The patients with longer field lengths appear to be the most relevant for a closer examination of the margins (mainly for the lumbar isocenter) or alternatively, a more comprehensive imaging protocol can be applied for these patients, such as daily IGRT. Based on our results, IGRT generally, and IGRT (wj) specifically is the superior choice for these patients. Centers that do not have this option should investigate their margins according to these uncertainties, especially for longer field lengths and higher number of isocenters.

Like previous studies [31, 32], we found that applying any type of imaging protocol reduces the uncertainties and residual setup errors compared to only using skin-marks for patient alignment. This difference was smaller for patients treated in the earlier years. Both imaging protocols and immobilizations have changed over the years, which affects this trend. In the era of daily image guidance, the difference might also originate from less time being spent on patient alignment when a verification image is pending.

When investigating the isocenters individually, the positioning errors and uncertainties found in this study are comparable to previously published research for other sites [8, 13–15]. Al-Wassia et al. [33] studied the effect of a 3-DoF couch correction, and found uncertainties that were substantially lower than ours for the single isocenter treatment. Their maximum mean deviation, in any direction, was found to be 6 mm while ours was 24 mm. Our results were, however, comparable to other similar studies investigating errors, uncertainties and margins for craniospinal treatments [34–36]. Stoiber et al. [34] found a maximum deviation of 18 mm and 10 °, again compared to our 24 mm and 9.6 °. Gupta et al. [35] found a maximum deviation of 20 mm. Interestingly, Thondykandy et al. [36] found the SU to be larger than the RU for CSI while our results show the opposite. The SE was investigated as an additional control to check that there were no systematic setup errors occurring in our imaging that potentially could bias the results, and indeed we found a SE close to zero.

## Conclusions

Our results show that daily IGRT substantially reduces setup uncertainties for pediatric CSI patients. Following a daily IGRT-protocol does, however, not guarantee satisfactory alignment when only a 3-DoF couch shift is applied. There are still quite large residual errors, some of which are the result of using multiple isocenters and narrow field junctions even if a 6-DoF couch shift would be applied. In conclusion, daily IGRT is the superior choice for setup of pediatric craniospinal patients, however, for centers that do not have this option, these results could be used to improve their margins and uncertainties for a more accurate treatment or used as a reference when comparing setup verification techniques.

## Data Availability

The datasets generated and/or analyzed during the current study are not publicly available due to that individual privacy could be compromised. Some restrictions apply to the availability of this data and parts of the data could become available from the corresponding author on reasonable request and with permission of PMR and KN.

## Abbreviations

CSI: Craniospinal irradiation
IGRT: Image-guided radiotherapy
AL: Action-level
NAL: Non-action level
DoF: Degrees of freedom
CNS: Central nervous system
OARs: Organs-at-risk
PTV: Planning target volume
MVCT: Mega-voltage computed tomography
kV: Kilo-voltage
CBCT: Cone-beam computed tomography
SI: Superior-inferior
AP: Anterior-posterior
ML: Medial-lateral
SD: Standard deviation
SE: Systematic error
SU: Systematic uncertainty
RU: Random uncertainty
BMI: Body mass index
nj: Narrow field junctions
wj: Wide field junctions
VMAT: Volumetric modulated arc therapy
IMPT: Intensity modulated proton therapy
